# Disrupted autophagy after spinal cord injury is associated with ER stress and neuronal cell death

**DOI:** 10.1038/cddis.2014.527

**Published:** 2015-01-08

**Authors:** S Liu, C Sarkar, M Dinizo, A I Faden, E Y Koh, M M Lipinski, J Wu

**Affiliations:** 1 Department of Orthopaedics, University of Maryland School of Medicine, Baltimore, MD, USA; 2 Department of Anesthesiology, Shock, Trauma and Anesthesiology Research (STAR) Center, University of Maryland School of Medicine, Baltimore, MD, USA; 3 Department of Anatomy and Neurobiology, University of Maryland School of Medicine, Baltimore, MD, USA

## Abstract

Autophagy is a catabolic mechanism facilitating degradation of cytoplasmic proteins and organelles in a lysosome-dependent manner. Autophagy flux is necessary for normal neuronal homeostasis and its dysfunction contributes to neuronal cell death in several neurodegenerative diseases. Elevated autophagy has been reported after spinal cord injury (SCI); however, its mechanism, cell type specificity and relationship to cell death are unknown. Using a rat model of contusive SCI, we observed accumulation of LC3-II-positive autophagosomes starting at posttrauma day 1. This was accompanied by a pronounced accumulation of autophagy substrate protein p62, indicating that early elevation of autophagy markers reflected disrupted autophagosome degradation. Levels of lysosomal protease cathepsin D and numbers of cathepsin-D-positive lysosomes were also decreased at this time, suggesting that lysosomal damage may contribute to the observed defect in autophagy flux. Normalization of p62 levels started by day 7 after SCI, and was associated with increased cathepsin D levels. At day 1 after SCI, accumulation of autophagosomes was pronounced in ventral horn motor neurons and dorsal column oligodendrocytes and microglia. In motor neurons, disruption of autophagy strongly correlated with evidence of endoplasmic reticulum (ER) stress. As autophagy is thought to protect against ER stress, its disruption after SCI could contribute to ER-stress-induced neuronal apoptosis. Consistently, motor neurons showing disrupted autophagy co-expressed ER-stress-associated initiator caspase 12 and cleaved executioner caspase 3. Together, these findings indicate that SCI causes lysosomal dysfunction that contributes to autophagy disruption and associated ER-stress-induced neuronal apoptosis.

In the United States, spinal cord injury (SCI) has an annual incidence of 11 000 and prevalence of nearly 500 000. Neuronal cell death is an important contributor to SCI-induced neurological deficits. Many of the affected neurons do not die because of direct mechanical damage but rather show delayed cell death as a result of injury-induced biochemical changes (secondary injury).^1, 2, 3, 4^ Thus, blocking or attenuating secondary neuronal death may serve to limit posttraumatic disabilities.

Macroautophagy (hereafter called autophagy) is a lysosome-dependent catabolic pathway degrading cytoplasmic proteins, protein aggregates and organelles.^5, 6, 7^ Autophagy is initiated by the formation of autophagosomes, double membrane vesicles containing cytoplasmic components that include potentially toxic protein aggregates and damaged organelles. Autophagosomes then fuse with lysosomes to allow degradation of their contents by lysosomal hydrolases.^8, 9, 10, 11^ This progress of cargo, from sequestration in autophagosomes, to their delivery and degradation in lysosomes, is termed autophagy flux. Autophagy flux is important for homeostasis in all cells but appears especially critical in terminally differentiated cells such as neurons.^12, 13^ It is also upregulated, and often plays a protective function, in response to cell injury.^14, 15^ For example, autophagy is activated in response to and can limit effects of homeostasis perturbation in the endoplasmic reticulum (ER stress).^16, 17^ Thus, autophagy plays an important neuroprotective function, while impaired autophagy flux has been implicated in neurodegenerative disorders such as Parkinson's and Alzheimer's diseases.^18, 19, 20, 21^

Upregulation of autophagy markers has been observed after SCI,^22, 23^ but its mechanisms and function remain controversial, with both beneficial and detrimental roles proposed. Under certain circumstances, pathologically increased autophagy can contribute to cell death,^21, 24^ particularly when autophagy flux is blocked, for example, because of lysosomal defects. Defects in autophagy flux can also exacerbate ER stress and potentiate ER-stress-induced apoptosis.^16, 17^ ER stress has long been implicated as part of the secondary injury after central nervous system trauma,^25, 26^ but its mechanisms remain unknown.

In the current study, we characterized the temporal distribution and cell-type specificity of autophagy following contusive SCI in a rat model. Our data demonstrate that autophagosome accumulation after SCI is not due to increased initiation of autophagy, but rather due to inhibition of autophagy flux. This likely reflects the disruption of lysosomal function after SCI. Pathological accumulation of autophagosomes is prominent in ventral horn (VH) motor neurons, where it is associated with signs of ER stress and related apoptosis. Together, our findings suggest that autophagy is disrupted after SCI and may exacerbate ER stress and neuronal cell death.

## Results

### Autophagosomes accumulate after SCI because of impaired autophagy flux

**Figure 1:**
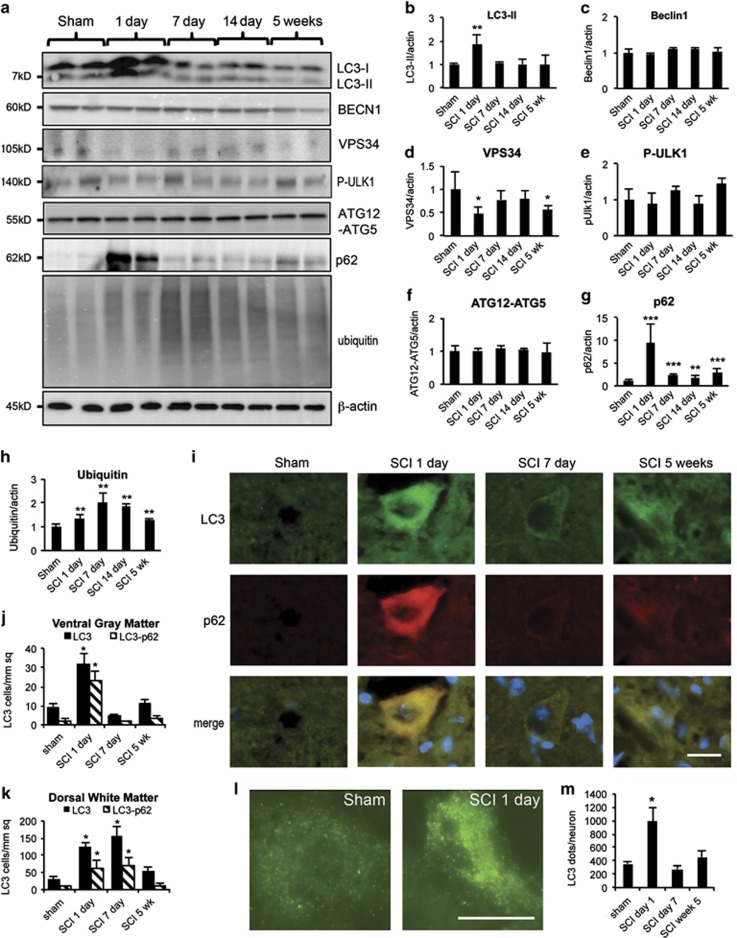
Accumulation of autophagosomes after SCI is due to impaired autophagy flux. (**a**) Representative western blots of autophagy markers LC3, Beclin 1, VPS34, phospho-ULK1 (P-ULK1), ATG12-ATG5 conjugate, p62 and ubiquitinated proteins in lysates from spinal cord of sham and SCI rats. Each lane represents an individual animal. (**b**–**h**) Quantification of western blot data from **a**. Data represent mean±S.D. normalized to corresponding sham; *n*≥4; *P<0.05, **P<0.01, ***P<0.001 by one-way ANOVA, followed by two-tailed *t*-test. (**i**) Representative images ( × 20) of IHC staining against LC3 (green) and p62 (red) in VH of gray matter of sham and SCI animals. Co-localization of LC3 and p62 is apparent at day 1 after SCI. Scale bar is 20 *μ*m. (**j** and **k**) Quantification of LC3-positive and double-positive LC3^+^/p62^+^ cells in sham and SCI animals normalized to the total area imaged. (**l**) Representative high magnification images ( × 60) of LC3-positive cells in VH of gray matter from sham and day 1 SCI animals. Accumulation of LC3-positive puncta corresponding to autophagosomes is apparent after SCI. Scale bar is 10 *μ*m. (**m**) Quantification of LC3-positive puncta in VH of gray matter normalized to the total number of imaged cells with neuronal morphology. All IHC data represent mean±S.E.; *n*≥4; *P<0.05 by one-way ANOVA, followed by *post hoc*

To examine the induction of autophagy after SCI, we assessed levels of autophagy protein LC3 (MAP1LC3B) in the injured spinal cord by western blot. Conversion of LC3-I to LC3-II by the addition of phosphatidylethanolamine is essential for the formation of autophagsomes and is considered a marker of autophagosome formation and accumulation. Levels of LC3-II significantly increased at day 1 after injury (*P*<0.01), then decreased by day 7 (Figures 1a and b), indicating accumulation of autophagosomes soon after injury. To confirm that LC3-II associated with autophagosomal membranes, we performed subcellular fractionation of injured and sham spinal cord. LC3-II accumulated primarily in the crude lysosomal/heavy membrane fraction (marked with LAMP1, Supplementary Figure S1a and c), confirming translocation to the membrane.

To investigate the mechanisms of autophagy after SCI, we examined levels of proteins involved in the regulation and formation of autophgosomes. The type III PI3 kinase, (including catalytic subunit VPS34 (PIK3C3) and regulatory protein Beclin1 (BECN1)) and the ULK1 complex are involved in the initiation of autophagy. In addition, autophagy protein 12 and 5 (ATG12-ATG5) conjugation is necessary for autophagosome elongation. Levels of BECN1, phospho-ULK1 and ATG12-ATG5 were not significantly altered, whereas VPS34 was slightly decreased after SCI (Figures 1a and c–f), suggesting that initiation of autophagy is not increased.

Increased accumulation of LC3-II could be due to either increased formation of autophagosomes or their decreased degradation. The adapter protein p62 (SQSTM1) mediates delivery of ubiquitinated cargo to autophagosomes. As p62 is degraded by autophagy alongside its cargo, accumulation of p62 indicates disrupted autophagic degradation. Levels of p62 increased immediately after injury, with a peak at day 1 (*P*<0.001). p62 levels decreased by day 7 but remained above baseline for at least 5 weeks after SCI compared with sham mice (Figures 1a and g). Therefore, initial accumulation of LC3-II after SCI likely reflects decrease in autophagy flux. Ubiquitinated proteins can be degraded by autophagy as well as the proteasome. Levels of ubiquitinated proteins gradually increased beginning at day 1 (*P*<0.01) and peaked at day 7 (*P*<0.01). They started decreasing from day 14 but remained above sham levels for at least 5 weeks (Figures 1a and h). These findings are consistent with a general defect in protein degradation after SCI, including decreased autophagy flux.

To confirm these findings, we performed immunohistochemistry (IHC) co-staining with antibodies against LC3 and p62 1 mm rostral to the epicenter. Quantitative image analysis was performed independently in four anatomical regions: dorsal horn (DH), VH, dorsal column and ventral medial white matter (Supplementary Figure S2). Consistent with the western blot data, the overall numbers of both LC3-positive cells and p62-positive cells were significantly higher in both gray (Figures 1i and j, and Supplementary Figure S3a and d) and white matter (Figure 1k and Supplementary Figure S4a and e) of the spinal cord starting at day 1 after injury as compared with sham. In the white matter, LC3- and p62-positive cells were most prominent on the dorsal side, close to the injury site. In the gray matter, LC3- and p62-positive cells preferentially accumulated in the VH. At high magnification ( × 60), we observed accumulation of LC3-positive puncta corresponding to autophagosomes (Figures 1l and m). After day 7, the number of both LC3- and p62-positive cells decreased toward sham levels (Figure 1j and Supplementary Figure S3b and d). Importantly, LC3 and p62 signals strongly co-localized at day 1 after injury (Figures 1j and k and Supplementary Figure S3d and S4e), suggesting that the same cells accumulated autophagosomes and p62. Specifically, 73% of LC3-positive cells were p62-positive in the VH of gray matter (Figure 1j), 63% in DH (Supplementary Figure S3d), 74% in the ventral white matter (Supplementary Figure S4e) and 51% in the dorsal white matter (Figure 1k). These data indicate that autophagosomes accumulate after SCI because of the disrupted clearance. In both ventral and DH, numbers of LC3-positive cells rose slightly at 5 weeks after injury (Figure 1j and Supplementary Figure S3d), whereas levels of p62 remained low (Supplementary Figure S3b and c). This suggests that initiation and flux of autophagy may be increased late after SCI.

### Lysosomal dysfunction may contribute to the disruption of autophagy flux

**Figure 2:**
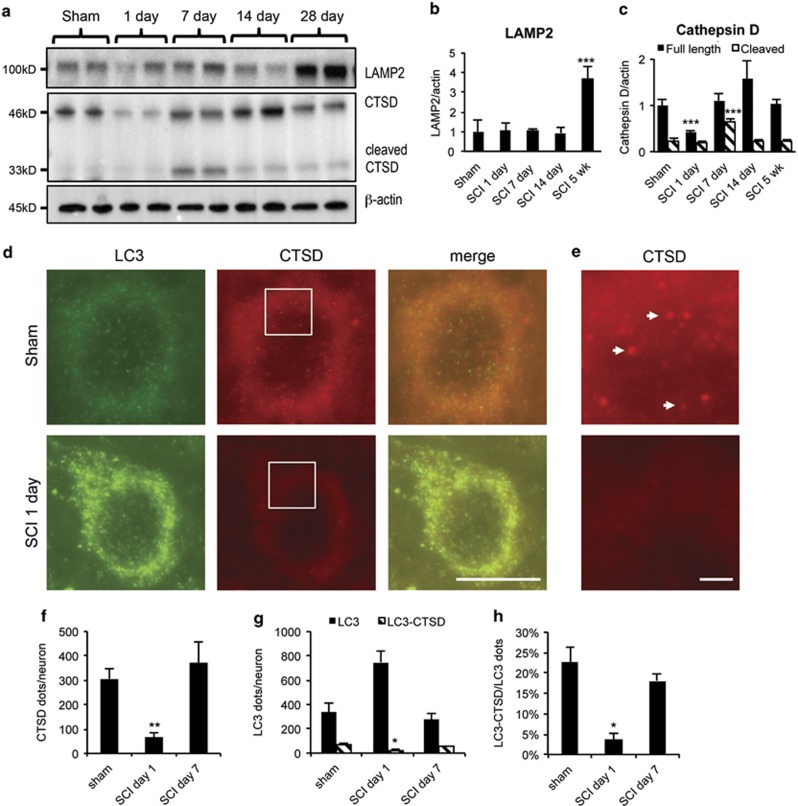
SCI leads to lysosomal dysfunction. (**a**) Representative western blots of lysosomal proteins LAMP1 and CTSD from spinal cord of sham and SCI animals. (**b** and **c**) Quantification of western blot data from **a**. Data represent mean±S.D. normalized to corresponding sham; *n*≥4; ***P<0.001 by one-way ANOVA, followed by two-tailed *t*-test. (**d**) Representative high magnification images ( × 60) of LC3 (green) and CTSD (red) staining in VH of gray matter from sham and day 1 SCI animals. Decrease in number and intensity of CTSD puncta (lysosomes) and increase in LC3 puncta (autophagosomes) is apparent after SCI. Scale bar is 10 *μ*m. (**e**) Close-up of the indicated area (white box) from CTSD staining in (**d**). Arrows point to representative CTSD-positive lysosomes. Scale bar is 1 *μ*m. (**f**–**h**) Quantification of data from (**d**): (**f**) CTSD-positive puncta (lysosomes), (**g**) LC3-positive puncta (autophagosomes) and LC3^+^/CTSD^+^ double-positive puncta (autolysosomes) in VH of gray matter normalized to the total number of imaged cells with neuronal morphology. (**h**) Decrease in fraction of autolysosomes (LC3^+^/CTSD^+^) as compared with all autophagosomes (LC3^+^) at day 1 after SCI. All IHC data represent mean±S.E.; *n*≥3; *P<0.05, **P<0.01 by one-way ANOVA, followed by *post hoc*

Under physiological conditions, lysosomes fuse with autophagosomes to allow degradation of autophagosomal cargo by lysosomal hydrolases. Therefore, impaired lysosomal function could lead to the disruption of autophagy flux. To investigate lysosomal function after SCI, we assessed the expression of lysosomal proteins by western blot. Levels of the membrane-associated lysosomal protein LAMP2 remained constant at days 1–14 after SCI (Figures 2a and b), suggesting that the size of the lysosomal compartment was not altered. In contrast, levels of both full-length cathepsin D (CTSD) and processed CTSD declined at day 1 after SCI as compared with sham (Figures 2a and c). Levels of CTSD recovered by day 7 (Figures 2a and c), correlating with restoration of autophagy flux. Levels of LAMP2 did not increase until day 28 after SCI (Figures 2a and b).

We used high-resolution imaging to assess co-localization of lysosomes (marked with CTSD) with autophagosomes (marked with LC3). Consistent with the western blot data, we detected significant decrease in the number and intensity of CTSD-positive lysosomes at day 1 after injury (Figures 2d and f), with recovery by day 7. At day 1, we also observed decreased LC3^+^/CTSD^+^ autolysosomes, suggesting decreased autophagosome–lysosome fusion (Figures 2g and h). The numbers of autolysosomes recovered by day 7 after injury. Together, these data suggest that the lysosomal protease levels and thus lysosomal function are impaired at day 1 after SCI (Figures 2d and f), contributing to disruption of fusion and clearance of autophagosomes. At day 7, increased CTSD levels may support recovery of autophagic flux.

### Autophagosomes accumulate in neurons, activated microglia and oligodendrocytes

**Figure 3:**
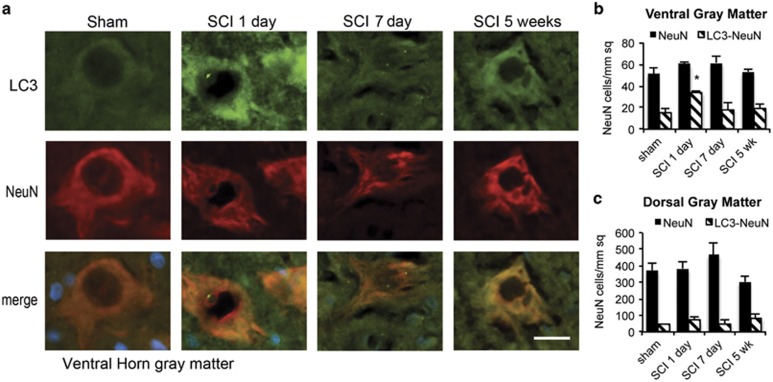
Autophagosomes accumulate in neurons of the VH gray matter at day 1 after SCI. (**a**) Representative images of IHC staining for LC3 (green) and neuronal marker NeuN (red) in VH of gray matter from sham and SCI animals. Stronger co-localization between NeuN and LC3 is apparent at day 1 after SCI. Scale bar is 20 *μ*m. (**b** and **c**) Quantification of neurons (NeuN+) co-localizing with LC3 in ventral (**b**) and dorsal (**c**) horn of gray matter from sham and SCI animals normalized to total area imaged. Data are normalized to total area imaged and represent mean±S.E.; *n*≥4; *P<0.05, by one-way ANOVA, followed by *post hoc*

In order to determine cell-type specificity of autophagosome accumulation, we performed IHC co-staining using antibodies against LC3 and different cell-type markers. In the VH, which contains motor neurons, we detected above-background levels of LC3 in many NeuN (RBFOX3)-positive neurons even in sham animals. Additional LC3 accumulation occurred mostly in neurons, with 57% of NeuN-positive neurons LC3 positive at day 1 after SCI (*P*<0.05, Figures 3a and b). LC3 induction in the DH of gray matter, which contains sensory neurons, was weaker (Figure 3c and Supplementary Figure S5). Although some NeuN-positive sensory neurons were positive for LC3, the co-localization was not as high (18–27%, Figure 3c). We did not observe significant co-localization of LC3 with any of the glial markers in the gray matter at any time points (Supplementary Figure S6a and d). Therefore, within the gray matter, NeuN-positive neurons preferentially accumulate autophagosomes 1 day after SCI, with VH motor neurons particularly affected. Together with the high LC3 and p62 co-localization at this time, this suggests that autophagy flux is disrupted after SCI in VH motor neurons.

**Figure 4:**
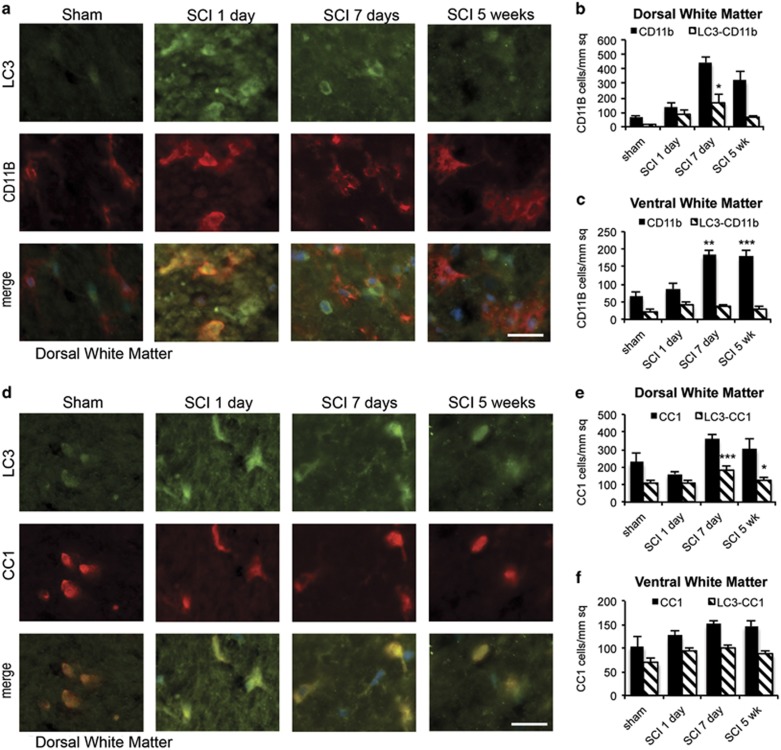
Autophagosomes accumulate in microglia and oligodendrocytes of the dorsal white matter adjacent to injury after SCI. (**a**) Representative images of IHC staining for LC3 (green) and activated microglia marker CD11B (red) in dorsal white matter of sham and SCI animals. Increased co-localization between LC3 and CD11B is apparent at day 1 after SCI. (**b** and **c**) Quantification of microglia (CD11B^+^) co-localizing with LC3 in dorsal (**b**) and ventral (**c**) white matter from sham and SCI animals. (**d**) Representative images of IHC staining for LC3 (green) and oligodendrocytes marker CC1 (red) in dorsal white matter of sham and SCI animals. Increased co-localization between LC3 and CC1 is apparent after SCI. All scale bars are 20 *μ*m. (**e** and **f**) Quantification of oligodendrocytes (CC1^+^) co-localizing with LC3 in dorsal (**e**) and ventral (**f**) white matter from sham and SCI animals. Data are normalized to total area imaged and represent mean±S.E.; *n*≥4; *P<0.05, **P<0.01, ***P<0.001 by one-way ANOVA, followed by *post hoc*

In the white matter, the total number of LC3-positive cells was most significantly increased near the injury site in the dorsal column (Figure 1k and Supplementary Figure S4a). In this region, total number of LC3positive cells peaked at 7 days after SCI and declined but remained above sham levels at 5 weeks. The absolute number of LC3-positive activated microglia (marked with cluster of differentiation 11b (CD11B)) in the dorsal white matter also peaked at day 7 (*P*<0.05, Figures 4a and b). Despite higher total numbers, the degree of co-localization of LC3 and CD11B was highest at 1 day rather than 7 days after injury (65% *versus* 38%, respectively, Figure 4b). A similar, albeit less pronounced, trend was observed in the ventral medial white matter (48% and 20% at days 1 and 7, respectively, Figure 4c and Supplementary Figure S7a). On the basis of cell morphology, LC3 accumulated preferentially in the most activated ameboid CD11B-positive cells (Figures 4a and b). These findings suggest that after SCI, autophagosomes may transiently accumulate in newly activated CD11B-positive microglia. As co-localization between LC3 and p62 is high at day 1, this is likely due to disrupted autophagic degradation.

In addition to microglia, we also observed the accumulation of LC3 in adenomatosis polyposis coli (*Apc*) gene product (clone CC1) (CC1)-positive oligodendrocytes. Similarly to motor neurons, many oligodendrocytes had above-background levels of LC3 even in sham animals (46% and 67% co-localization of LC3 and CC1 in dorsal and ventral white area, respectively (Figures 4d–f and Supplementary Figure S7b). Increased proportion of CC1-positive cells co-localized with LC3 in dorsal white matter 1 day after SCI (69%, Figure 4e), whereas total numbers of LC3^+^/CC1^+^ cells peaked at day 7 (*P*<0.001). A similar trend was detected in the ventral white matter (75% co-localization at day 1, Figure 4f and Supplementary Figure S7b). The level of co-localization returned to base-line level at day 35 (Figures 4e and f).

Unlike microglia and oligodendrocytes, the number of LC3-positive astrocytes (marked with glial fibrillary acid protein (GFAP)) remained very low in all regions of the white matter at all time points examined (Supplementary Figure S8a and d), suggesting that levels of autophagy were not significantly altered in astrocytes after SCI.

### Disruption of autophagy flux is associated with neuronal cell death

**Figure 5:**
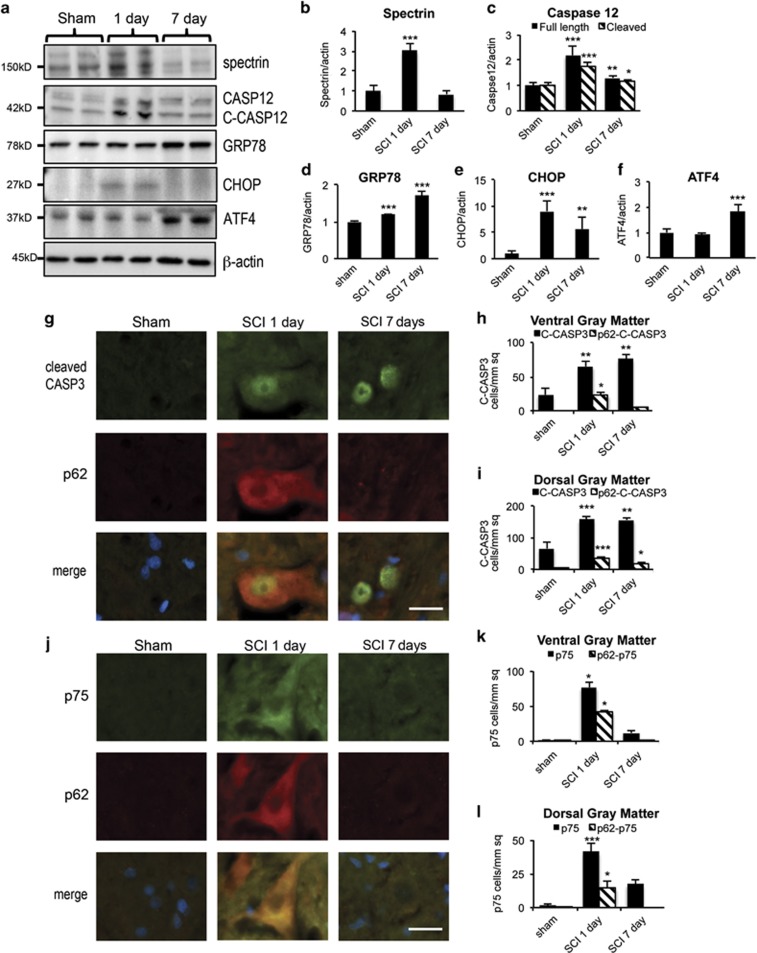
Impaired autophagy flux is associated with neuronal cell death after SCI. (**a**) Representative western blots of cell death (spectrin, caspase 12 (CASP12)) and ER stress (GRP78, CHOP, ATF4) markers from spinal cord of sham and SCI animals. (**b–f**) Quantification of western blot data from **a**. Data represent mean±S.D. normalized to corresponding sham; *n*≥4; *P<0.05, **P<0.01, ***P<0.001 by one-way ANOVA, followed by two-tailed *t*-test. (**g**) Representative images of IHC staining for cleaved caspase 3 (C-CASP3, green) and p62 (red) in VH of gray matter from sham and SCI animals. Co-localization between cleaved CASP3 and p62 is apparent at day 1 after SCI. (**h** and **i**) Quantification of cleaved CASP3-positive cells and double-positive C-CASP3^+^/p62^+^ cells in ventral (**f**) and dorsal (**g**) horns of gray matter from sham and SCI animals. (**j**) Representative images of IHC staining for p75 (green) and p62 (red) in VH of gray matter from sham and SCI animals. Co-localization is apparent at day 1 after SCI. All scale bars are 20 *μ*m. (**k** and **l**) Quantification of p75-positive and double-positive p75^+^/p62^+^ cells in ventral (**i**) and dorsal (**j**) horns of gray matter from sham and SCI animals. All IHC data are normalized to total area imaged and represent mean±S.E.; *n*≥4; *P<0.05, **P<0.01, ***P<0.001 by one-way ANOVA, followed by *post hoc*

Disruption of autophagy flux contributes to neuronal cell death in many neurodegenerative diseases.^27, 28^ Therefore, we investigated the relationship between disrupted degradation of autophagosomes and neuronal cell death after SCI. The levels of breakdown products of spectrin (145–150 kDa), a marker of cell death, peaked at day 1 after SCI (*P*<0.001), declining to background levels by day 7 (Figures 5a and b). This was mirrored by the accumulation of both full-length and cleaved caspase 12 (CASP12, *P*<0.001), a marker of ER-stress-induced apoptosis (Figures 5a and c). Therefore, cell death markers peaked at day 1 after SCI, a time point correlating with the maximal accumulation of p62, suggesting a potential causative relationship between disruption of autophagy and neuronal cell death.

To investigate the specific role of disrupted autophagy flux in neuronal cell death, we quantified co-localization of cell death markers with p62 in the gray matter of the spinal cord by IHC staining. Caspase 3 (CASP3), the major executioner caspase, is cleaved to an active form during apoptosis. The numbers of cleaved CASP3-postive cells in both horns of the gray matter were greatly increased by day 1, and remained elevated at day 7 after SCI (*P*<0.01 at day 1 and 7 in all regions, Figures 5g–i and Supplementary Figure S9a). In the ventral gray matter at day 1, 35% of cleaved CASP3-positive cells were also positive for p62 (Figure 5h), indicating that the impairment of autophagy flux correlated with induction of apoptosis. The relationship was weaker in the DH, where 22% of cleaved CASP3-positive cells co-stained with p62 (Figure 5i and Supplementary Figure S9a). In both regions, co-localization of cleaved CASP3 and p62 declined considerably by day 7 (Figures 5h and i). Similar results were obtained using antibodies against the low affinity nerve growth factor receptor (NGFR/p75), which is known to accumulate in apoptotic neurons after SCI. Induction of p75 was most pronounced in the VH neurons where it strongly co-localized with p62 (*P*<0.05, 54%, Figures 5j and k). p75 staining and its co-localization with p62 was much weaker in the DH (35%, Figure 5i and Supplementary Figure S9b).

**Figure 6:**
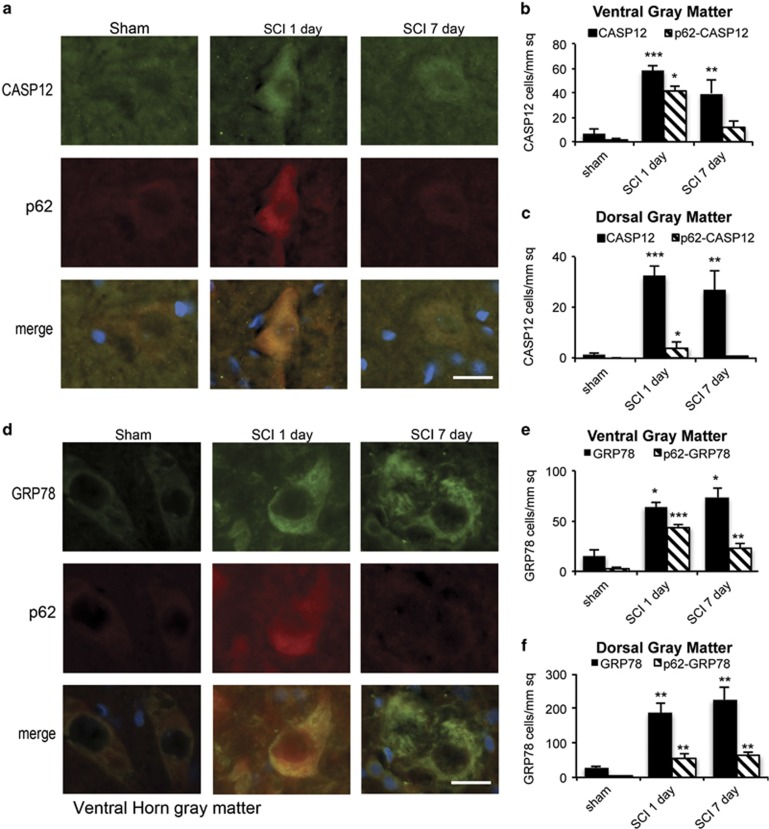
Impaired autophagy flux is associated with ER stress and ER-stress-induced neuronal apoptosis after SCI. (**a**) Representative images of IHC staining for ER-stress-associated caspase 12 (CASP12, green) and p62 (red) in VH of the gray matter from sham and SCI animals. Co-localization of CASP12 and p62 is apparent at day 1 after SCI. (**b** and **c**) Quantification of CASP12-positive and CASP12^+^/p62^+^ double-positive cells in ventral (**b**) and dorsal (**c**) horns of gray matter from sham and SCI animals. (**d**) Representative images of IHC staining for ER stress marker GRP78 (green) and p62 (red) in VH of gray matter from sham and SCI animals. Co-localization of GRP78 and p62 is apparent at 1 day after SCI. All scale bars are 20 *μ*m. (**e** and **f**) Quantification of GRP78-positive and GRP78^+^/p62^+^ double-positive cells in ventral (**e**) and dorsal (**f**) horns of gray matter from sham and SCI animals. Data are normalized to total area imaged and represent mean±S.E.; *n*≥4; *P<0.05, **P<0.01, ***P<0.001 by one-way ANOVA, followed by *post hoc*

Next, we examined the expression of ER-stress-associated CASP12. ER stress can be influenced by autophagy,^25, 29^ which is thought to be a part of cytoprotective UPR response to accumulation of misfolded proteins in the ER lumen.^17^ ER stress is induced and involved in mediation of secondary injury after SCI,^30^ but its mechanisms are not known. The numbers of CASP12-positive cells in the gray matter peaked at day 1 after SCI and declined sharply by day 7 (Figures 6a–c and Supplementary Figure S10a). The majority of positive cells had neuronal morphology. Although the overall number of apoptotic (cleaved CASP3-positive) cells was much higher in the dorsal than in the VH (Figures 5h and i), CASP12-positive cells were more prevalent in the ventral than in the dorsal gray matter (Figures 6a–c and Supplementary Figure S10a). There was also much higher co-localization of CASP12 and p62 signal in the VH (70% *versus* 21%, *P*<0.05 for both, Figures 6b and c). These data suggest that defect in autophagy flux observed in the VH may specifically contribute to and exacerbate ER stress and consequently CASP12-dependent apoptosis in motor neurons.

To directly evaluate the possibility that defects in autophagy flux may contribute to ER stress observed after SCI, we examined the expression of ER stress markers, the 78 kDa glucose-regulated protein (GRP78) and transcription factors ATF4 and CHOP (DDIT3). Western blot analysis revealed that GRP78 and CHOP were significantly increased by 1 day after injury (*P* <0.001, Figures 5a, d and e). GRP78 as well as ATF4 continued to accumulate for at least 7 days (*P*<0.001, Figures 5a, d and f), whereas CHOP, which is involved in mediation of ER-stress-induced apoptosis, declined. Expression of GRP78 in the gray area was confirmed by IHC analysis (Figures 6d–f). In the VH, 68% of GRP78-positive cells were also positive for p62 (Figure 6e) indicating defective autophagy flux. The majority of the affected cells had neuronal morphology. Although GRP78 staining further increased at day 7 after injury, co-localization with p62 declined (Figure 6e). Increased numbers of GRP78-positive cells were present also in the DH; however, co-localization with p62 was much weaker at all time points (under 30%, Figure 6f and Supplementary Figure S10b). Together, our data indicate a strong association of defects in autophagy flux with induction of ER stress and ER-stress-mediated cell death in VH neurons after SCI.

## Discussion

Disruption of autophagy flux has been reported to contribute to neuronal cell death in neurodegenerative diseases, such as Parkinson disease, Alzherimer's disease and Huntington disease.^5, 31, 32^ Although elevated autophagy markers have been detected after SCI, its mechanism, its cell-type specificity and its relationship with cell death remained unknown. In the current study, we demonstrate that several upstream regulators and mediators of autophagy remain unchanged or even decline after SCI. Therefore, increased initiation of autophagy cannot account for the observed accumulation of autophagosomes. Instead, as indicated by the accumulation of autophagy substrates, the apparent elevation of autophagy appears because of disruption of autophagosome degradation. This is associated with decrease in cathepsin-D-positive lysosomes and autolysosomes, suggesting that lysosomal defects after SCI may cause impairment of autophagosome–lysosome fusion and autophagy flux. Accumulation of p62 has been reported following chronic mechanical compression of the spinal cord.^23^ However, neither autophagy flux nor lysosomal function has been investigated after acute SCI. Thus, our data for the first time identify the cellular mechanism leading to the accumulation of autophagosomes after SCI.

In the present study, we also identify the specific times, regions and cell types in which autophagosomes accumulate after SCI. In the white matter, microglia and oligodendrocytes are preferentially affected. The extent of autophagosome accumulation correlates with the proximity to the injury site, with the dorsal column showing greatest changes. In the gray matter, the accumulation of autophagosomes occurs primarily in neurons and is more pronounced in the VH motor neurons as compared with the DH sensory neurons. Co-localization between p62, a marker of inhibited autophagy flux, and neuronal cell death markers is also greater in the VH. Therefore, the normal function of motor neurons may be particularly dependent on autophagy flux, making these cells more vulnerable to its disruption.

Autophagy is essential for neuronal cell health and survival, and its defects can directly lead to neurodegeneration *in vivo*.^12, 13^ The mechanisms include accumulation of toxic protein aggregates and defective organelles.^27, 28^ Accumulated autophagosomes themselves may also serve a pathologic function.^27, 33^ As the initial accumulation of autophagosomes in neuronal cells occurs at a time when autophagy is blocked, it likely contributes to neuronal cell death. This is supported by a strong co-localization of p62 with markers of cell death including cleaved caspase 3, caspase 12 and p75 in cells with neuronal morphology. Therefore, our data are consistent with a model where lysosomal damage-induced autophagy dysfunction contributes to neuronal cell death after SCI. Dysfunctional lysosomes have been detected in neurodegenerative diseases, including Parkinson's disease, and in lysosomal storage diseases, where they cause defects in autophagy flux and contribute to neurodegeneration.^19, 20, 34, 35, 36^ Therefore, our data also suggest a potential common mechanism of neuronal cell death caused by both chronic (neurodegenerative and lysosomal storage diseases) and acute (SCI) insults.

Activation of ER stress and its role in secondary injury after SCI have been previously reported.^25, 26^ The mechanisms contributing to ER stress after SCI are, however, unknown. Autophagy is commonly induced and can serve as a protective mechanism in response to ER stress.^16, 17^ Our data demonstrate that increased ER stress and ER-stress-induced apoptosis strongly correlate with attenuated autophagy flux in VH neurons. Therefore, the inhibition of autophagy flux after SCI may contribute to neuronal ER stress. Markers of ER stress remain increased at day 7, a time when autophagy flux is starting to be restored. However, at that time, they are no longer associated with induction of caspase 12 and CHOP, markers of ER-stress-induced apoptosis. We postulate that the inhibition of autophagy flux may exacerbate ER stress in motor neurons, contributing to the induction of apoptosis. Consistently, although ER stress is also apparent in dorsal sensory neurons, induction of caspase 12 is much less pronounced and correlates less well with defects in autophagy flux.

Accumulation of p62 and to some extent ubiquitinated proteins resolves at later time points (starting by day 7) after SCI, suggesting recovery of autophagy flux. This could be due to the death of cells with blocked autophagy flux. However, because decrease in p62 correlates with increased CTSD expression, improved lysosomal function could eventually allow restoration of autophagy flux in at least some of the affected cells. This should return autophagy to its normal neuroprotective function and could contribute to the spontaneous locomotor recovery usually observed around day 7 after SCI. Although treatments known to alter levels of autophagy can influence SCI outcomes,^37, 38^ their effects on lysosomal function or autophagy flux have not been evaluated. Additionally, each of the drugs used has multiple functions and it is not known whether their effects on SCI were mediated via autophagy. For example, rapamycin, an inhibitor of mTOR complex 1 (mTORC1) and an inducer of autophagy, improved SCI outcomes in mice.^37^ However, the inhibition of mTOR not only induces autophagy, but can also enhance lysosomal biogenesis.^39, 40^ Our data suggest that functional improvements induced by rapamycin after SCI^37, 41^ could be mediated, at least partially, by the stimulation of lysosomal biogenesis after mTOR inhibition, leading to early restoration of autophagy flux. We postulate that other drugs that enhance lysosomal function or biogenesis may also represent potential treatments after SCI.

## Materials and Methods

### Contusive SCI

A well-documented procedure of moderate SCI contusion was performed in adult male Sprague-Dawley rats.^42, 43, 44^ Briefly, after rats (235–275 g) were anesthetized with sodium pentobarbital (65 mg/kg intraperitoneal injection), a laminectomy was performed at T8 to remove the part of the vertebra overlying the spinal cord, exposing a circle of dura. A moderate contusion injury was produced by dropping a 10 g weight from 2.5 cm onto an impounder positioned on the exposed spinal cord without disrupting the dura. Body temperature was kept by maintaining the animal on a heating pad (37 °C) throughout the procedure. After SCI, rats were kept on highly absorbent bedding and their bladders manually expressed twice daily until a reflex bladder was established (7–14 days after SCI). Sham rats were subjected to anesthesia and a laminectomy but were not injured. All experiments complied fully with the principles set forth in the ‘Guide for the Care and Use of Laboratory Animals' and were approved by the University of Maryland Animal Care and Use Committee.

### Western blot analysis

Rat spinal cord tissue (5 mm) centered on the injury site were lysed and homogenized in RIPA buffer (Sigma-Aldrich Co. LLC, St. Louis, MO, USA) supplemented 1x protease inhibitor cocktail (Sigma-Aldrich Co. LLC), phosphatase inhibitor cocktail II and III (Sigma-Aldrich Co. LLC), sonicated and centrifuged at 20 000 *g* for 20 min. Protein concentrations were determined by the Pierce BCA method (ThermoFisher Scientific, Waltham, MA, USA). Samples were run on 4–15% or 15% SDS-PAGE (Bio-Rad, Hercules, CA, USA), and transferred to PVDF membrane (Bio-Rad). Membranes were incubated with primary antibodies overnight, with HRP-conjugated secondary antibodies for 1 h, visualized using SuperSignal West Dura Extended Duration Substrate (ThermoFisher Scientific), and imaged with ChemIDoc TM MP system (Bio-Rad). The signal was quantified by Image Lab software (Bio-Rad). Primary antibodies: LC3 (1 : 200, Novus, Cat. No. NB100-2220, Littleton, CO, USA), p62 (1 : 200; Progen, Cat. No. GP62-C, Heidelberg, Germany), Beclin1 (1:1000; Santa Cruz Biotechnology, Cat. No. sc-11427, Dallas, TX, USA), phospho-ULK1 (1:1000; Cell Signaling, Cat. No. 5869, Danvers, MA, USA), VPS34 (1 : 1000, Sigma-Aldrich, V9764), ATG5 (1 : 1000; Sigma-Aldrich, Cat. No. A0731), *β*-actin (1:10,000; Sigma-Aldrich, Cat. No. A1978), Ubiquitin (1 : 1000; Cell Signaling, Cat. No. 3936), CTSD (1 : 100, Santa Cruz Biotechnology, Cat. No. sc-6486), Spectrin (1 : 5000; Enzo Life Science International, Cat. No. BML-FG6090, Farmingdale, NY, USA), cleaved caspase 3 (1 : 500, Cell Signaling, Cat. No. 9661), caspase 12 (1 : 200, Cell Signaling, Cat. No. 2202), GRP78 (1 : 500, Cell Signaling, Cat. No. 3177), CHOP (1 : 1000, Cell Signaling, Cat. No. 2895), ATF4 (1 : 1000, Sigma Aldrich, Cat. No. WH0000468M1), and LAMP2 (1 : 1000, Cat No. ABL-93) (developed by J. Thomas August and obtained from Developmental Studies Hybridoma Bank developed under the auspices of the NICHD and maintained by The University of Iowa, Department of Biology, Iowa City, IA 52242, USA).

### Subcellular fractionation

Five millimeter fragments of spinal cord tissue centered on the injury site were collected from rats at day 1 after injury and homogenized in ice-cold buffered solution containing 0.32 M sucrose, 10 mM Hepes and protease and phosphatase inhibitors. Homogenates were centrifuged at 800 *g* for 10 min at 4 °C to pellet down the nuclei. Supernatants were sequentially cenrifuged at 20 000* g* for 20 min at 4 °C to pellet the heavy membrane/crude lysosomal fractions and at 100 000 *g* for 1 h at 4 °C to pellet light membrane fractions. All pellets were re-suspended in homogenization buffer and protein concentration was estimated using BCA reagent (ThermoFisher Scientific). Fractions were then analyzed by western blot.

### Immunohistochemistry

Animals were intracardially perfused with PBS, then with 4% paraformaldehyde. A 1.0-cm segment of spinal cord centered at the injury epicenter was sectioned at 20-*μ*m thickness and thaw-mounted onto Superfrost Plus slides (Fisher Scientific, Pittsburgh, PA, USA). Sections were blocked in 5% goat or donkey serum in PBS/0.025% Triton X-100, incubated with primary antibodies overnight and with secondary antibodies for 1 h. Cell nuclei were labeled with 4',6-diamidino-2-phenylindole (Sigma-Aldrich Co. LLC), slides were coverslipped with an anti-fading medium (Hydromount; National Diagnostics, Atlanta, GA, USA). Primary antibodies: LC3 (1 : 200, Novus, Cat. No. NB100-2220), p62 (1 : 200; Progen, Cat. No. GP62-C), NeuN (1 : 500; Millipore, Cat. No. MAB377, Billerica, MA, USA), CD11b (1 : 500, Abcam, Cat. No. ab8878, Cambridge, MA, USA), CC1 (1 : 1000, Abcam, Cat. No. ab16794), GFAP (1 : 1000; Dako, Cat. No. Z0334, Carpinteria, MA, USA), CTSD (1 : 100, Santa Cruz Biotechnology, Cat. No. sc-6486), cleaved caspase 3 (1 : 500, Cell Signaling, Cat. No. 9661), caspase 12 (1 : 200, Cell Signaling, Cat. No. 2202), GRP78 (1 : 500, Abcam, Cat. No. ab21685), and p75 (1 : 500, Cell Signaling, Cat. No. 2693). Secondary antibodies: alexa fluor 488 goat anti-rabbit (Cat. No. A11034), alexa fluor 546 goat anti-mouse (Cat. No. A11030), alexa fluor 568 goat anti-guinea pig (Cat. No. A11075), alexa fluor 633 goat anti-mouse (Cat. No. A21052) and alexa fluor 546 donkey anti-goat (Cat. No. A11056) (Invitrogen, Grand Island, NY, USA).

### Image acquisition and quantification

All images were acquired 2–3 mm rostral to the epicenter. Each cross section was divided in to four anatomical areas, and acquired images independently: (i) VH, (ii) DH, (iii) ventral medial white matter and (iv) dorsal column (Supplementary Figure S2). Initially, images were also acquired in the lateral white matter, however, because the observed phenotypes were intermediate between VMWM and DC, for the sake of simplicity, this area was not included in the final analysis. Three to five images per location per rat were acquired using a fluorescent Nikon Ti-E inverted microscope, at × 20 (CFI Plan APO VC 20 × NA 0.75 WD 1 mm) or × 60 (CFI Plan APO VC 60 × NA 1.4 Oil) magnification. All × 60 images were acquired as z-stacks and focused using Extended Depth of Focus module of Elements software (Nikon Inc., Melville, NY, USA). The background of each image was subtracted by using Elements. All images were quantified using Elements: nuclei were identified using Spot Detection algorithm; cells expressing LC3 or positive for any of the other immunofluorescence markers were identified using Detect Regional Maxima algorithm, followed by global thresholding. Number of positive cells was normalized to the total imaged area. Intracellular puncta were detected using Spot Detection and normalized to the number of neuronal cells imaged (determined by morphology). For each experiment, data from all images from one region in each rat were summed up and used for final statistical analysis. Approximately 1000 cells were quantified per rat per area per experiment.

### Statistics analysis

Unless indicated otherwise, results were expressed as mean±S.E.M., where ‘*n*' is the number of individual animals per group. All statistical analyses were conducted using SigmaPlot, Version 12 (Systat Software, San Jose, CA, USA) or GraphPad Prism, Version 3.02 for Windows (GraphPad Software, La Jolla, CA, USA). One-way ANOVA followed by Bonferroni, Tukey's or SNK *t*-test *post hoc* test were used for parametric data. Kruskal–Wallis ANOVA based on ranks and Dunn's *post hoc* test were used for non-parametric data. For experiments with only two groups, two-tailed Mann-Whitney Rank Sum Test (nonparametric) or two-tailed unpaired Student's *t*-test (parametric) was performed. A *P*-value≤0.05 was considered significant.

## Abbreviations

SCI: spinal cord injury
ER: endoplasmic reticulum
BECN1: beclin1
CTSD: cathepsin D
ATG5: autophagy protein 5
ATG12: autophagy protein 12
IHC: immunohistochemistry
LAMP2: lysosomal-associated membrane protein 2
CASP3: caspase 3
CASP12: caspase 12
NGFR: nerve growth factor receptor
GRP78: 78 kDa glucose-regulated protein
mTOR: mammalian target of Rapamycin
mTORC1: mTOR complex 1
T8: thoracic segment 8
RIPA: radioimmunoprecipitation
NeuN: neuronal nuclei/RNA binding protein, fox-1 homolog (C. elegans) 3
CD11B: cluster of differentiation 11b
CC1: adenomatosis polyposis coli (Apc) gene product (clone CC1)
GFAP: glial fibrillary acidic protein
VH: ventral horn
DH: dorsal horn

## References

[bib1] Dumont RJ, Okonkwo DO, Verma S, Hurlbert RJ, Boulos PT, Ellegala DB et al. Acute spinal cord injury, part I: pathophysiologic mechanisms. Clin Neuropharmacol 2001; 24: 254–264. 11586110 10.1097/00002826-200109000-00002

[bib2] Tator CH. Experimental and clinical studies of the pathophysiology and management of acute spinal cord injury. J Spinal Cord Med 1996; 19: 206–214. 9237787 10.1080/10790268.1996.11719436

[bib3] Grossman SD, Rosenberg LJ, Wrathall JR. Temporal-spatial pattern of acute neuronal and glial loss after spinal cord contusion. Exp Neurol 2001; 168: 273–282. 11259115 10.1006/exnr.2001.7628

[bib4] Beattie MS, Hermann GE, Rogers RC, Bresnahan JC. Cell death in models of spinal cord injury. Prog Brain Res 2002; 137: 37–47. 12440358 10.1016/s0079-6123(02)37006-7

[bib5] Yang Z, Klionsky DJ. Eaten alive: a history of macroautophagy. . Nat Cell Biol 2010; 12: 814–822. 20811353 10.1038/ncb0910-814 PMC3616322

[bib6] Caccamo A, Majumder S, Richardson A, Strong R, Oddo S. Molecular interplay between mammalian target of rapamycin (mTOR), amyloid-beta, and Tau: effects on cognitive impairments. . J Biol Chem 2010; 285: 13107–13120. 20178983 10.1074/jbc.M110.100420 PMC2857107

[bib7] Sarkar S, Floto RA, Berger Z, Imarisio S, Cordenier A, Pasco M et al. Lithium induces autophagy by inhibiting inositol monophosphatase. J Cell Biol 2005; 170: 1101–1111. 16186256 10.1083/jcb.200504035 PMC2171537

[bib8] Klionsky DJ, Emr SD. Autophagy as a regulated pathway of cellular degradation. Science 2000; 290: 1717–1721. 11099404 10.1126/science.290.5497.1717 PMC2732363

[bib9] Levine B, Klionsky DJ. Development by self-digestion: molecular mechanisms and biological functions of autophagy. Dev Cell 2004; 6: 463–477. 15068787 10.1016/s1534-5807(04)00099-1

[bib10] Mizushima N, Komatsu M. Autophagy: renovation of cells and tissues. Cell 2011; 147: 728–741. 22078875 10.1016/j.cell.2011.10.026

[bib11] Mizushima N. Autophagy in protein and organelle turnover. Cold Spring Harb Symp Quant Biol 2011; 76: 397–402. 21813637 10.1101/sqb.2011.76.011023

[bib12] Hara T, Nakamura K, Matsui M, Yamamoto A, Nakahara Y, Suzuki-Migishima R et al. Suppression of basal autophagy in neural cells causes neurodegenerative disease in mice. Nature 2006; 441: 885–889. 16625204 10.1038/nature04724

[bib13] Komatsu M, Waguri S, Chiba T, Murata S, Iwata J, Tanida I et al. Loss of autophagy in the central nervous system causes neurodegeneration in mice. Nature 2006; 441: 880–884. 16625205 10.1038/nature04723

[bib14] Hung SY, Huang WP, Liou HC, Fu WM. Autophagy protects neuron from Abeta-induced cytotoxicity. Autophagy 2009; 5: 502–510. 19270530 10.4161/auto.5.4.8096

[bib15] Xu Y, Yuan J, Lipinski MM. Live imaging and single-cell analysis reveal differential dynamics of autophagy and apoptosis. Autophagy 2013; 9: 1418–1430. 23748697 10.4161/auto.25080 PMC4026027

[bib16] Ogata M, Hino S, Saito A, Morikawa K, Kondo S, Kanemoto S et al. Autophagy is activated for cell survival after endoplasmic reticulum stress. Mol Cell Biol 2006; 26: 9220–9231. 17030611 10.1128/MCB.01453-06 PMC1698520

[bib17] Boyce M, Lipinski MM, Py BF, Yuan J. Endoplasmic reticulum stress response in cell death and cell survival In: Reed JC, Green DR (eds). Apoptosis: Physiology and Pathology vol. 1 Cambridge: Cambridge University Press, 2011; pp 51–62.

[bib18] Klionsky DJ. Neurodegeneration: good riddance to bad rubbish. Nature 2006; 441: 819–820. 16778876 10.1038/441819a

[bib19] Mizushima N, Levine B, Cuervo AM, Klionsky DJ. Autophagy fights disease through cellular self-digestion. Nature 2008; 451: 1069–1075. 18305538 10.1038/nature06639 PMC2670399

[bib20] Rubinsztein DC. The roles of intracellular protein-degradation pathways in neurodegeneration. Nature 2006; 443: 780–786. 17051204 10.1038/nature05291

[bib21] Nixon RA. Autophagy in neurodegenerative disease: friend, foe or turncoat? Trends Neurosci 2006; 29: 528–535. 16859759 10.1016/j.tins.2006.07.003

[bib22] Kanno H, Ozawa H, Sekiguchi A, Yamaya S, Itoi E. Induction of autophagy and autophagic cell death in damaged neural tissue after acute spinal cord injury in mice. Spine (Phila Pa 1976) 2011; 36: E1427–E1434. 21304420 10.1097/BRS.0b013e3182028c3a

[bib23] Tanabe F, Yone K, Kawabata N, Sakakima H, Matsuda F, Ishidou Y et al. Accumulation of p62 in degenerated spinal cord under chronic mechanical compression: functional analysis of p62 and autophagy in hypoxic neuronal cells. Autophagy 2011; 7: 1462–1471. 22082874 10.4161/auto.7.12.17892 PMC3288020

[bib24] Lipinski MM, Zheng B, Lu T, Yan Z, Py BF, Ng A et al. A genome-wide analysis reveals differential regulation of autophagy in normal brain aging and in Alzheimer's disease. Proc Natl Acad Sci USA. 2010; 107: 14164–14169. 20660724 10.1073/pnas.1009485107 PMC2922576

[bib25] Larner SF, Hayes RL, McKinsey DM, Pike BR, Wang KK. Increased expression and processing of caspase-12 after traumatic brain injury in rats. J Neurochem 2004; 88: 78–90. 14675152 10.1046/j.1471-4159.2003.02141.x

[bib26] Ohri SS, Hetman M, Whittemore SR. Restoring endoplasmic reticulum homeostasis improves functional recovery after spinal cord injury. Neurobiol Dis 2013; 58: 29–37. 23659896 10.1016/j.nbd.2013.04.021 PMC3748169

[bib27] Nixon RA. Autophagy, amyloidogenesis and Alzheimer disease. J Cell Sci 2007; 120(Pt 23): 4081–4091. 18032783 10.1242/jcs.019265

[bib28] Bove J, Martinez-Vicente M, Vila M. Fighting neurodegeneration with rapamycin: mechanistic insights. Nat Rev Neurosci 2011; 12: 437–452. 21772323 10.1038/nrn3068

[bib29] Tarabal O, Caldero J, Casas C, Oppenheim RW, Esquerda JE. Protein retention in the endoplasmic reticulum, blockade of programmed cell death and autophagy selectively occur in spinal cord motoneurons after glutamate receptor-mediated injury. Mol Cell Neurosci 2005; 29: 283–298. 15911352 10.1016/j.mcn.2005.03.003

[bib30] Nakagawa T, Zhu H, Morishima N, Li E, Xu J, Yankner BA et al. Caspase-12 mediates endoplasmic-reticulum-specific apoptosis and cytotoxicity by amyloid-beta. Nature 2000; 403: 98–103. 10638761 10.1038/47513

[bib31] Schaeffer V, Lavenir I, Ozcelik S, Tolnay M, Winkler DT, Goedert M. Stimulation of autophagy reduces neurodegeneration in a mouse model of human tauopathy. Brain 2012; 135(Pt 7): 2169–2177. 22689910 10.1093/brain/aws143 PMC3381726

[bib32] Rusten TE, Stenmark H. p62, an autophagy hero or culprit? Nat Cell Biol 2010; 12: 207–209. 20190829 10.1038/ncb0310-207

[bib33] Basit F, Cristofanon S, Fulda S. Obatoclax (GX15-070) triggers necroptosis by promoting the assembly of the necrosome on autophagosomal membranes. Cell Death Differ 2013; 20: 1161–1173. 23744296 10.1038/cdd.2013.45 PMC3741498

[bib34] Dehay B, Martinez-Vicente M, Caldwell GA, Caldwell KA, Yue Z, Cookson MR et al. Lysosomal impairment in Parkinson's disease. Mov Disord 2013; 28: 725–732. 23580333 10.1002/mds.25462 PMC5131721

[bib35] Vila M, Bove J, Dehay B, Rodriguez-Muela N, Boya P. Lysosomal membrane permeabilization in Parkinson disease. Autophagy 2011; 7: 98–100. 21045565 10.4161/auto.7.1.13933

[bib36] Shintani T, Klionsky DJ. Autophagy in health and disease: a double-edged sword. Science 2004; 306: 990–995. 15528435 10.1126/science.1099993 PMC1705980

[bib37] Sekiguchi A, Kanno H, Ozawa H, Yamaya S, Itoi E. Rapamycin promotes autophagy and reduces neural tissue damage and locomotor impairment after spinal cord injury in mice. J Neurotrauma 2012; 29: 946–956. 21806471 10.1089/neu.2011.1919

[bib38] Tang P, Hou H, Zhang L, Lan X, Mao Z, Liu D et al. Autophagy reduces neuronal damage and promotes locomotor recovery *via* inhibition of apoptosis after spinal cord injury in rats. Mol Neurobiol 2014; 49: 276–287. 23954967 10.1007/s12035-013-8518-3

[bib39] Pena-Llopis S, Vega-Rubin-de-Celis S, Schwartz JC, Wolff NC, Tran TA, Zou L et al. Regulation of TFEB and V-ATPases by mTORC1. EMBO J 2011; 30: 3242–3258. 21804531 10.1038/emboj.2011.257 PMC3160667

[bib40] Pena-Llopis S, Brugarolas J. TFEB, a novel mTORC1 effector implicated in lysosome biogenesis, endocytosis and autophagy. Cell Cycle 2011; 10: 3987–3988. 22101272 10.4161/cc.10.23.18251 PMC3272281

[bib41] Chen HC, Fong TH, Hsu PW, Chiu WT. Multifaceted effects of rapamycin on functional recovery after spinal cord injury in rats through autophagy promotion, anti-inflammation, and neuroprotection. J Surg Res 2013; 179: e203–e210. 22482761 10.1016/j.jss.2012.02.023

[bib42] Wu J, Raver C, Piao C, Keller A, Faden AI. Cell cycle activation contributes to increased neuronal activity in the posterior thalamic nucleus and associated chronic hyperesthesia after rat spinal cord contusion. Neurotherapeutics 2013; 10: 520–538. 23775067 10.1007/s13311-013-0198-1 PMC3701760

[bib43] Wu J, Stoica BA, Dinizo M, Pajoohesh-Ganji A, Piao C, Faden AI. Delayed cell cycle pathway modulation facilitates recovery after spinal cord injury. Cell Cycle 2012; 11: 1782–1795. 22510563 10.4161/cc.20153 PMC3372383

[bib44] Wu J, Stoica BA, Luo T, Sabirzhanov B, Zhao Z, Guanciale K et al. Isolated spinal cord contusion in rats induces chronic brain neuroinflammation, neurodegeneration, and cognitive impairment: Involvement of cell cycle activation. Cell Cycle 2014; 13: 15. 25483194 10.4161/cc.29420 PMC4128888

