# Supplementary material for: Disrupted autophagy after spinal cord injury is associated with ER stress and neuronal cell death
Source: Cell Death Dis. 2015 Jan 8;6(1):e1582–. doi: 10.1038/cddis.2014.527 (PMC4669738; doi:10.1038/cddis.2014.527)
Supplement: Supplementary Information [file cddis2014527x1.docx]

**Supplementary Information Summary**

1. Supplementary Figures: includes Supplementary Figures 1-10 and corresponding Figure Legends.
