# Supplementary material for: Disrupted autophagy after spinal cord injury is associated with ER stress and neuronal cell death
Source: Cell Death Dis. 2015 Jan 8;6(1):e1582–. doi: 10.1038/cddis.2014.527 (PMC4669738; doi:10.1038/cddis.2014.527)
Supplement: Supplementary Figures [file cddis2014527x2.pdf]

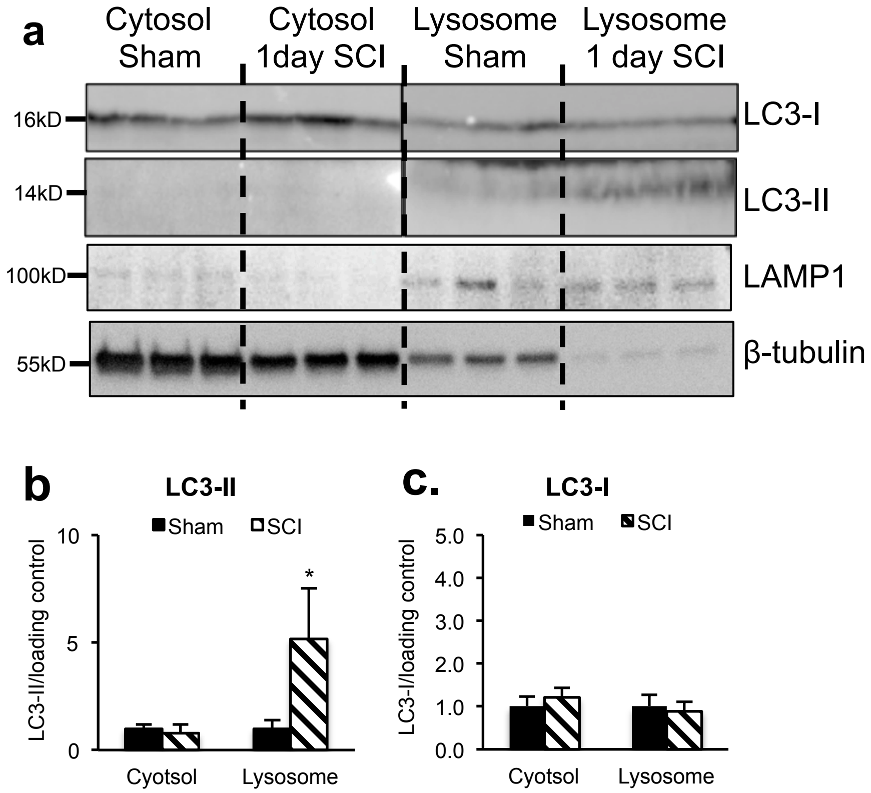

Supplementary Figure S1: LC3-II accumulates in lysosomal/heavy membrane fraction after SCI. **(a)** Sham and day 1 SCI spinal cords were fractionated to isolate cytosol and heavy membrane/lysosomal fractions; Levels of LC3 were assessed by western blot. b-tubulin and LAMP1 served as marker and loading controls in cytosolic and lysosomal fractions, respectively. **(b-c)** Quantification of western blot data from a. Data represent mean normalized to corresponding sham +/- SD; *p<0.05 by t-tailed student t-test.

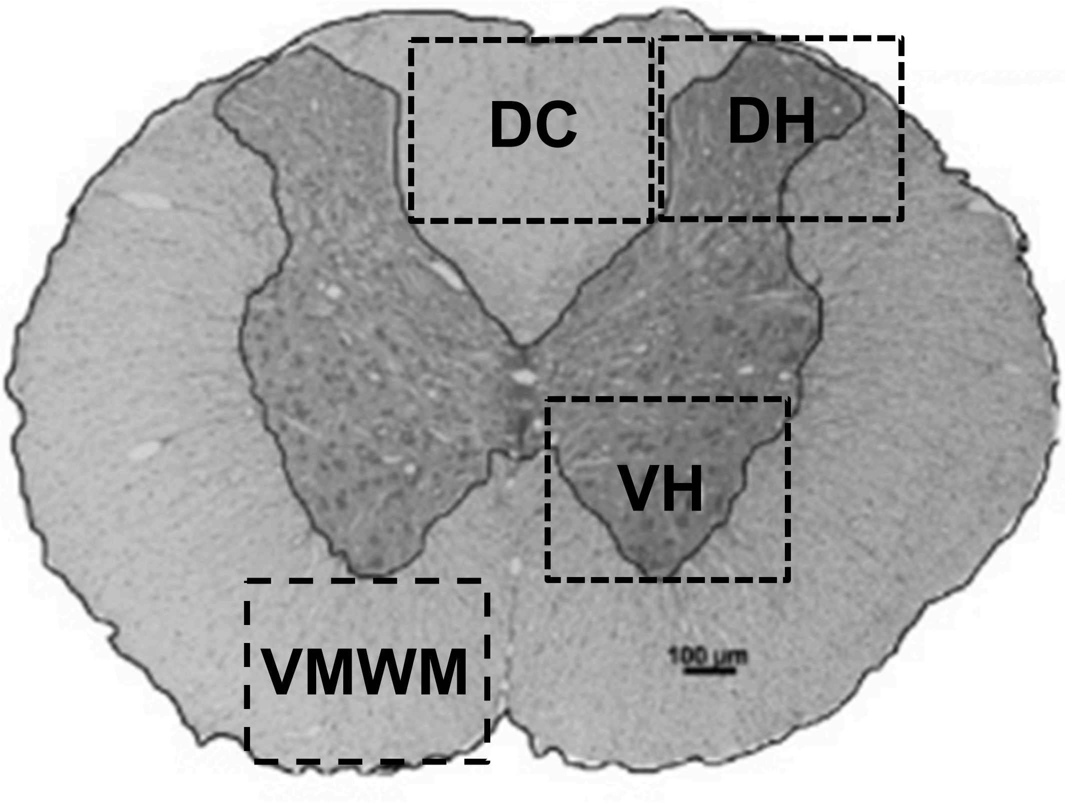

Supplementary figure S2: Illustration of the 4 anatomical regions used for image analysis. DH: dorsal horn; VH: ventral horn; DC: dorsal column; VMWM: ventral medial white matter. Dash line boxes indicate the region covered at 20x magnification.

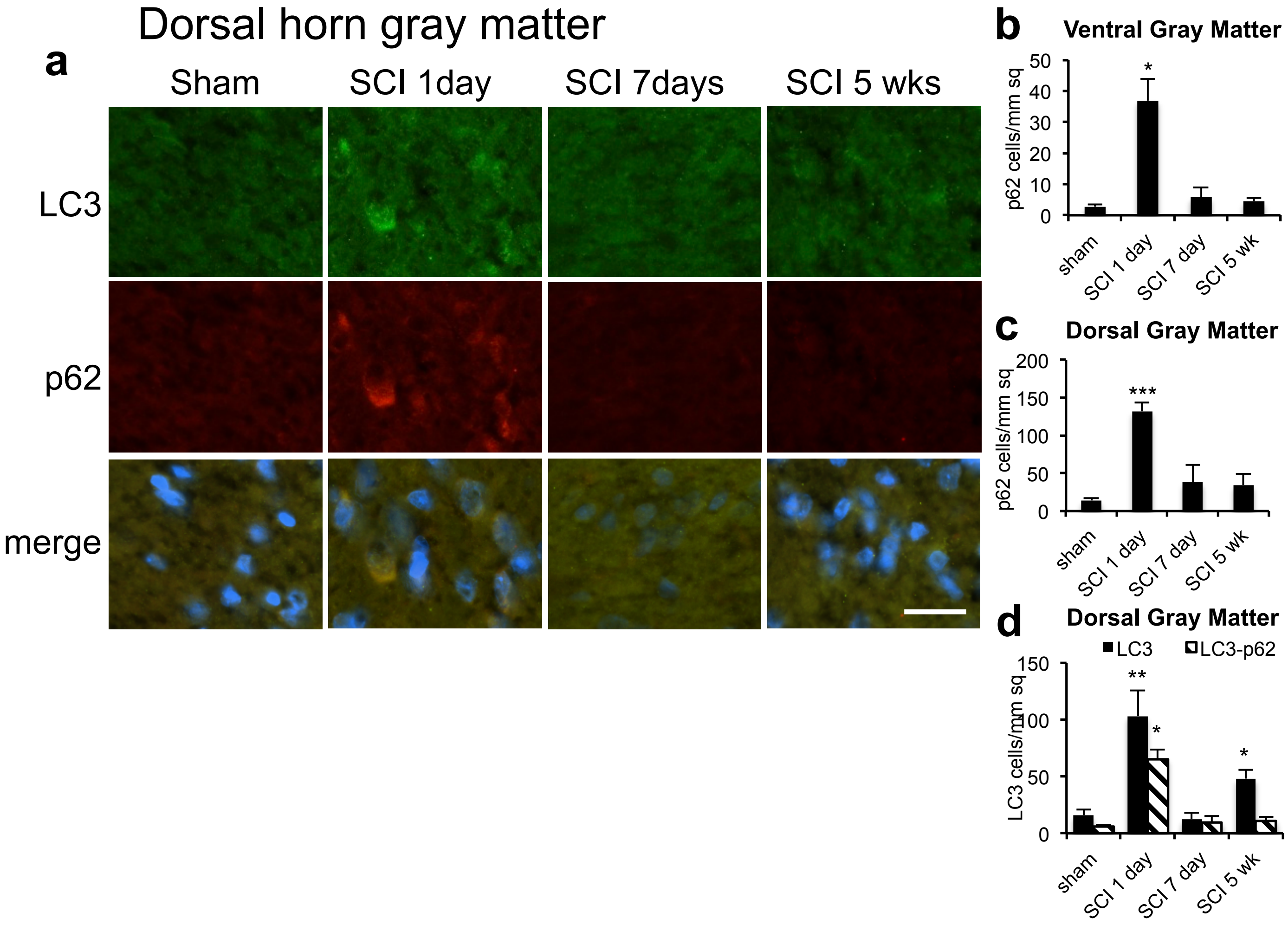

Supplementary Figure S3: LC3 and p62 are induced and co-localize in the gray matter after SCI. (a) Representative images of IHC staining for LC3 (green) and p62 (red) in the dorsal horn of sham and SCI animals. Scale bar is 20 μm. **(b-c)** Quantification of p62-positive cells in the gray matter from sham and SCI animals: **(b)** ventral horn, **(c)** dorsal horn. **(d)** Quantification of LC3 positive and LC3+/p62+ double positive cells in the dorsal horn after SCI. All data are normalized to total area imaged and represent mean +/- SE; n≥4; *p<0.05, **p<0.01, ***p<0.001 by one-way ANOVA, followed by post-hoc.

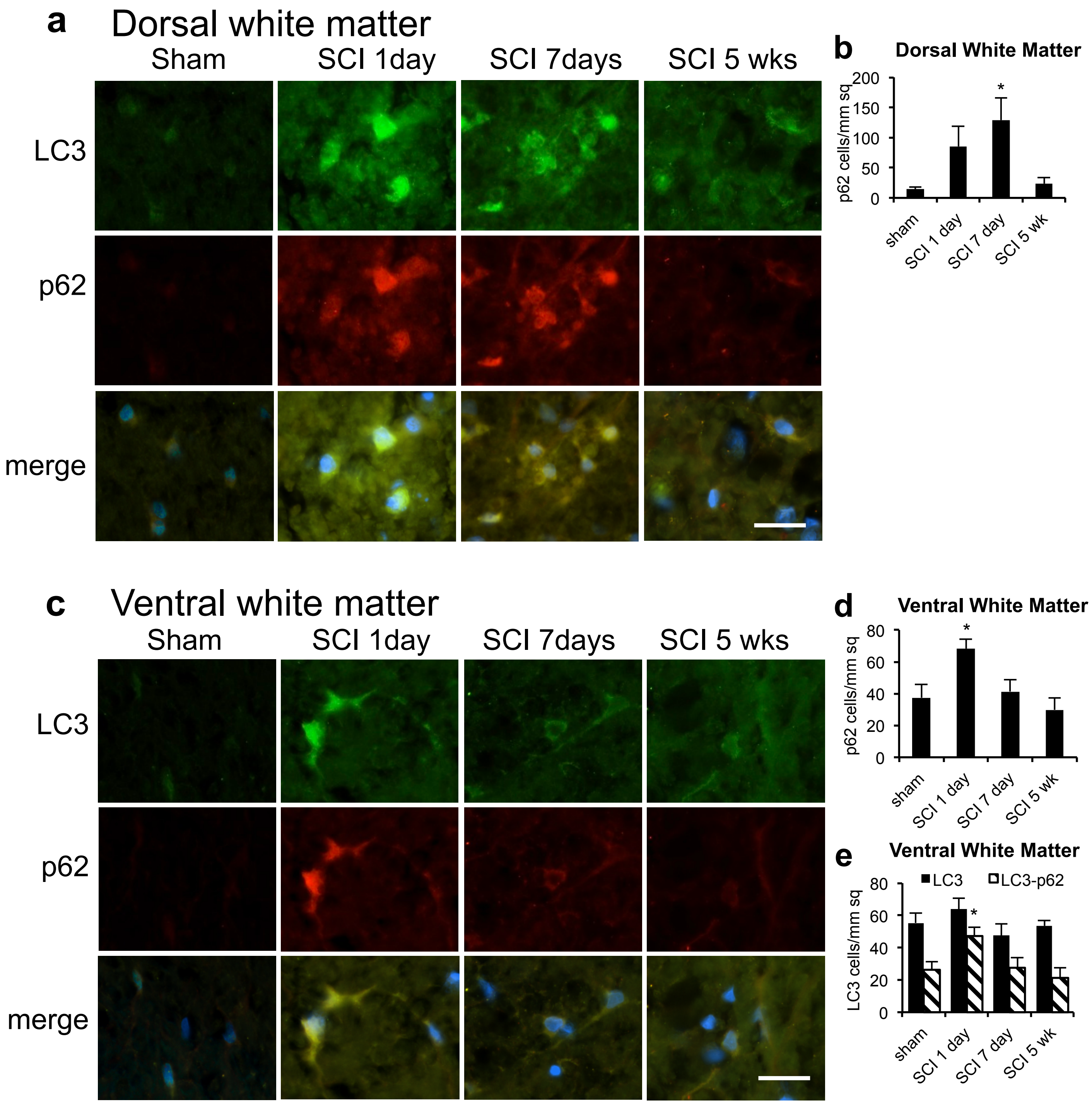

Supplementary Figure S4: LC3 and p62 are induced and co-localize in the white matter after SCI. (a) Representative images of IHC staining for LC3 (green) and p62 (red) in dorsal white matter (dorsal column) of sham and SCI animals. (b) Quantification of p62-positive cells in the dorsal white matter matter from sham and SCI animals. (c) Representative images of IHC staining for LC3 (green) and p62 (red) in ventral medial white matter of sham and SCI animals. All scale bars are 20 μm. (d) Quantification of p62-positive cells in the dorsal white matter matter from sham and SCI animals. (e) Quantification of LC3 positive and LC3+/p62+ double positive cells in ventral white matter after SCI. All data are normalized to total area imaged and represent mean +/- SE; n≥4; *p<0.05, **p<0.01, ***p<0.001 by one-way ANOVA, followed by post-hoc.

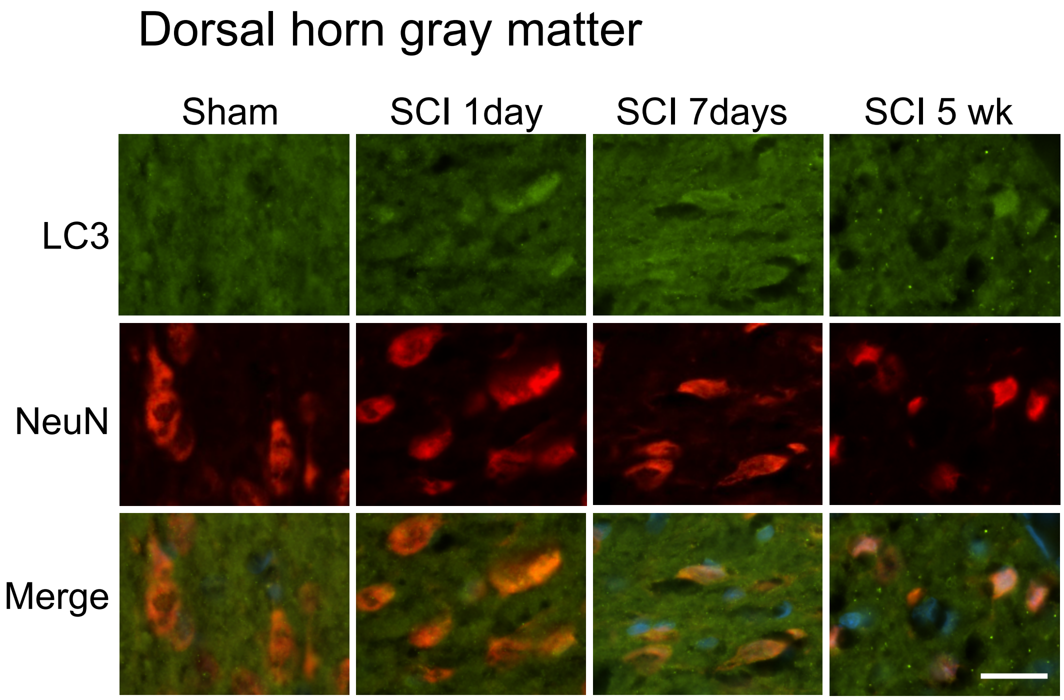

Supplementary Figure S5: Representative images of IHC staining for LC3 (green) and neuronal marker NeuN (red) in the dorsal horn from sham and SCI animals. Scale bar is 20 µm.

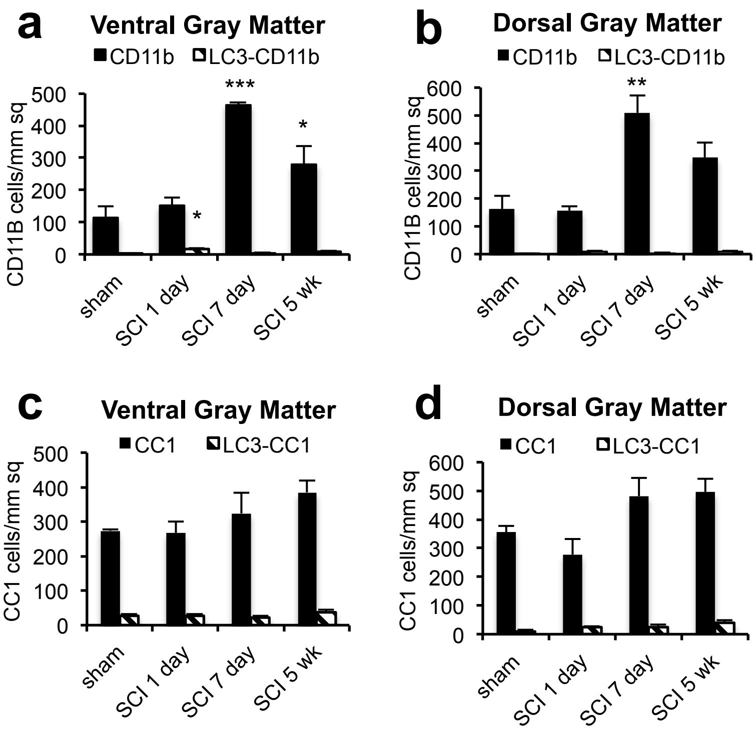

Supplementary Figure S6: Few autophagosomes accumulate in glial cells of the gray matter after SCI. **(a-b)** Quantification of microglia (CD11B+) co-localizing with LC3 in ventral (a) and dorsal (b) horn from sham and SCI animals. **(c-d)** Quantification of oligodendrocytes (CC1+) co-localizing with LC3 in ventral (c) and dorsal (d) horn from sham and SCI animals. Data area normalized to total area imaged and represent mean +/- SE; n≥4; *p<0.05, **p<0.01, ***p<0.001 by one-way ANOVA, followed by post-hoc.

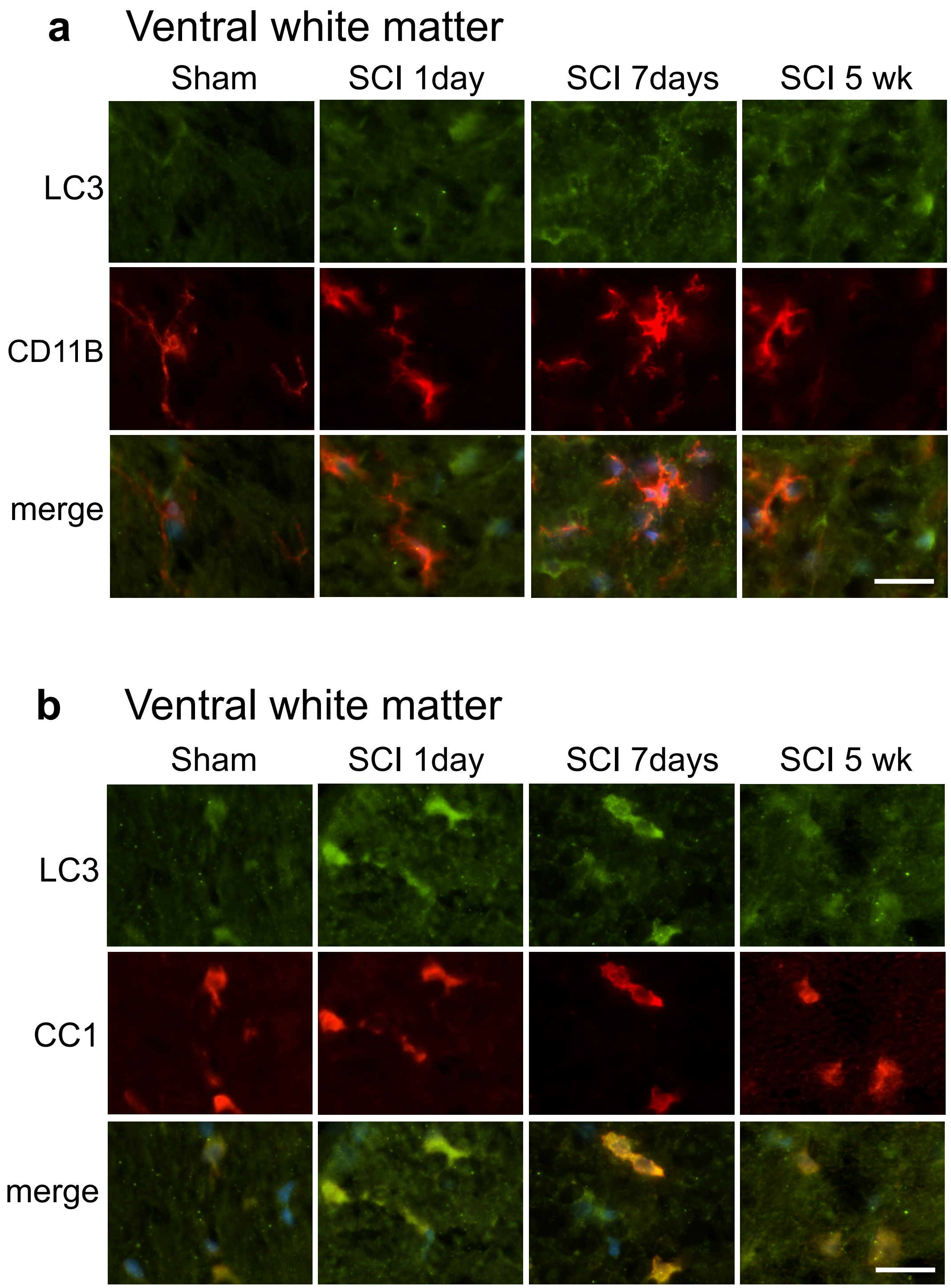

Supplementary Figure S7: Accumulation of autophagosomes in microglia and oligodendrocytes of ventral medial white matter after SCI. **(a)** Representative images of IHC staining for LC3 (green) and activated microglia marker CD11B (red) in ventral white matter of sham and SCI animals. **(b)** Representative images of IHC staining for LC3 (green) and oligodendrocytes marker CC1 (red) in ventral white matter of sham and SCI animals. Scale bars are 20 μm.

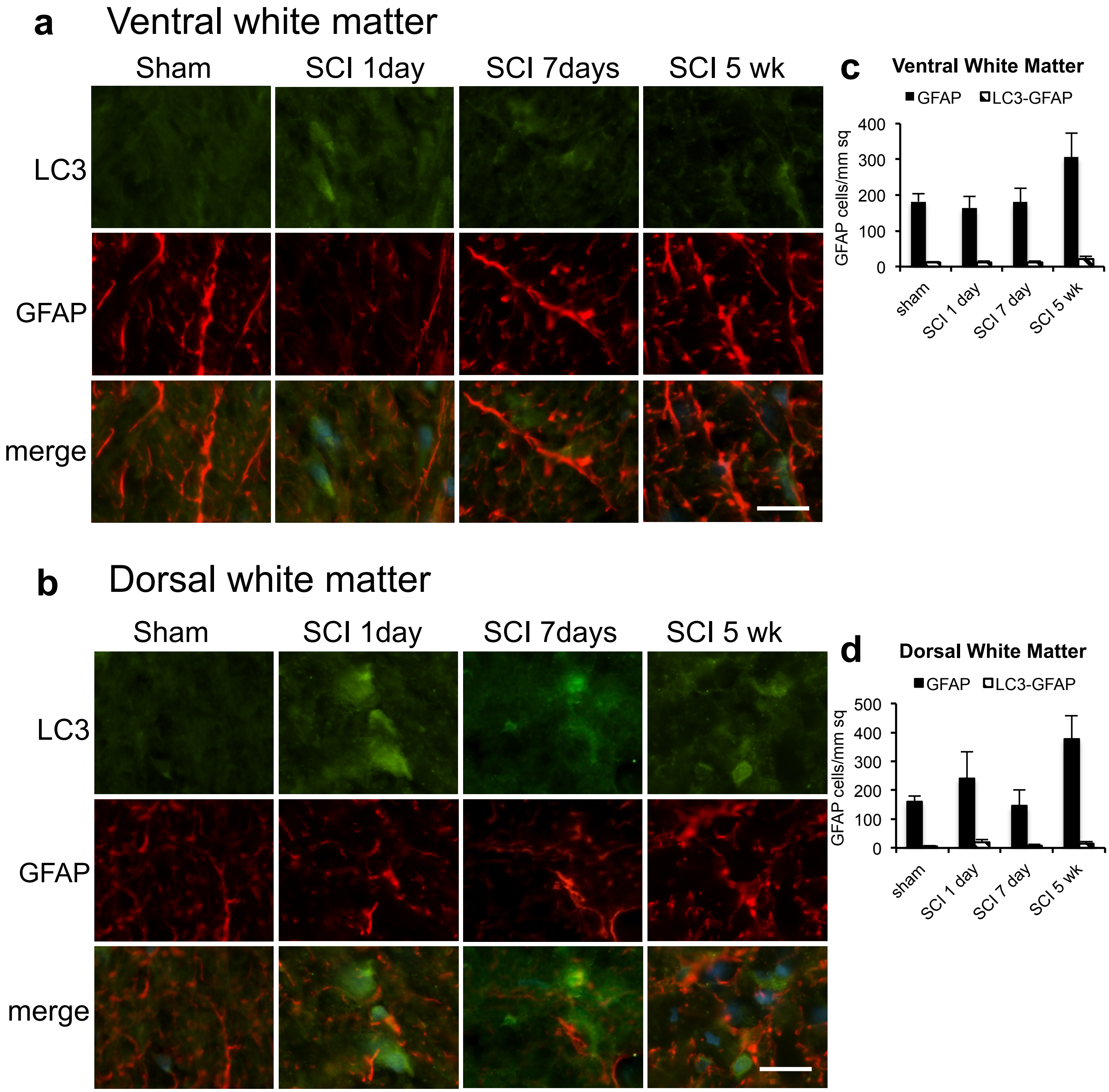

Supplementary Figure S8: Little accumulation of autophagosomes in astrocytes in the white matter after SCI. **(a-b)** Representative images of IHC staining for LC3 (green) and astrocyte marker GFAP (red) in ventral medial (a) and dorsal (b) white matter of sham and SCI animals. Scale bars are 20 μm. **(c-d)** Quantification of astrocytes (GFAP+) co-localizing with LC3 in ventral (c) and dorsal (d) white matter from sham and SCI animals. Data area normalized to total area imaged and represent mean +/- SE; n≥4

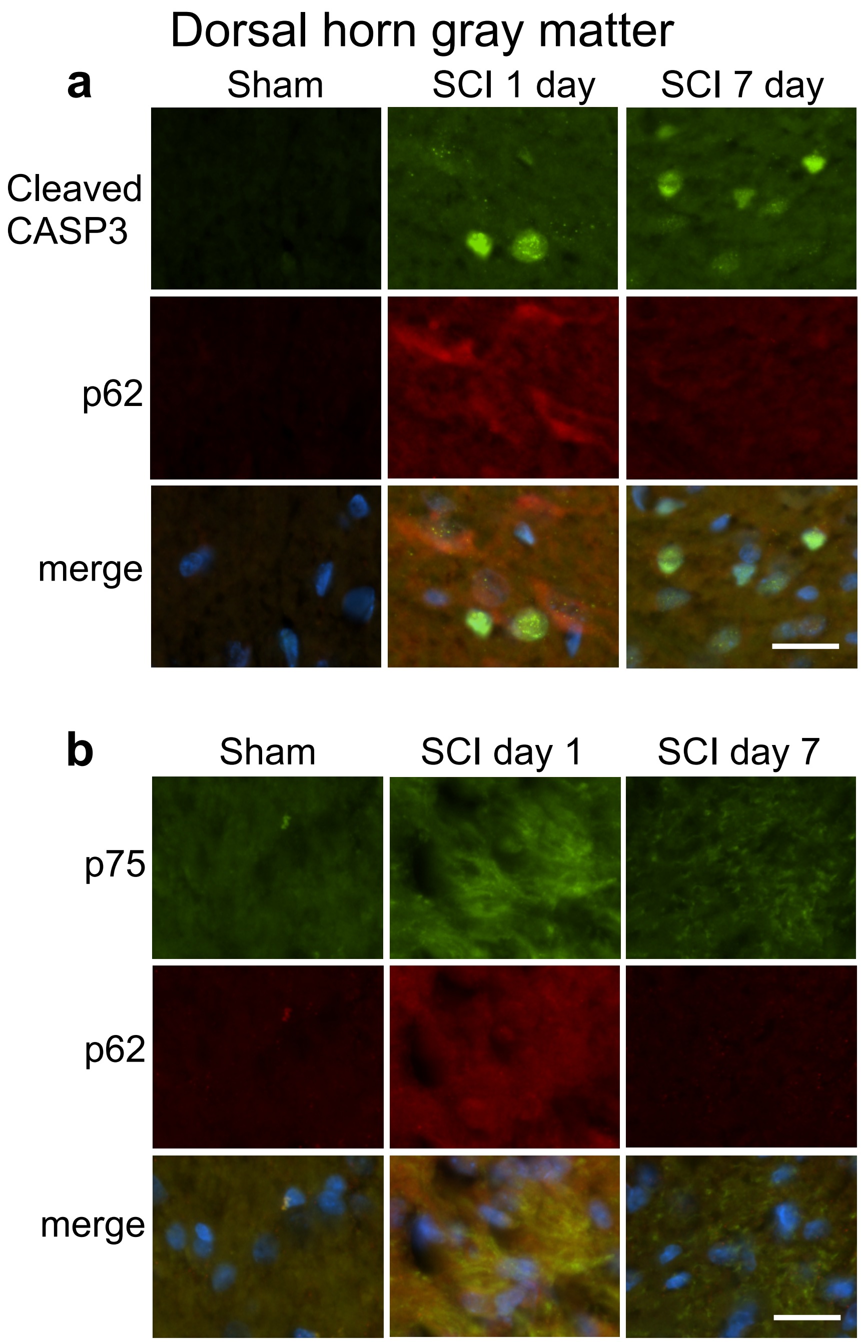

Supplementary Figure S9: Neuronal cell death is not associated with impaired autophagy flux in the dorsal horn after SCI. **(a)** Representative images of IHC staining for cleaved caspase 3 (CASP3, green) and p62 (red) in the dorsal horn from sham and SCI animals. **(b)** Representative images of IHC staining for p75 (green) and p62 (red) in the dorsal horn from sham and SCI animals. Scale bars are 20 μm.

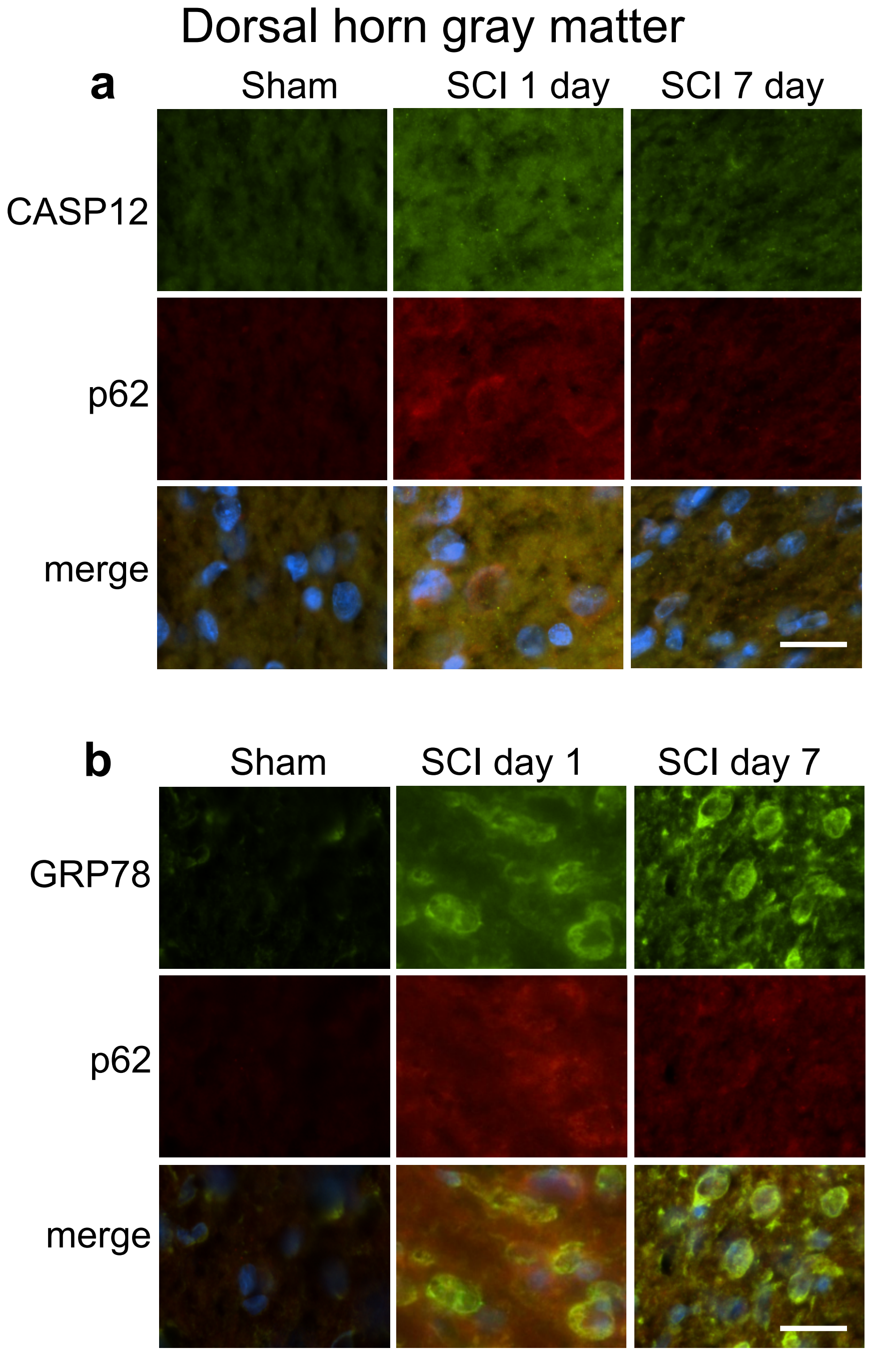

Supplementary Figure S10: ER-stress is not associated with impaired autophagy flux in the dorsal horn after SCI. **(a)** Representative images of IHC staining for caspase 12 (CASP12, green) and p62 (red) in the dorsal horn from sham and SCI animals. **(b)** Representative images of IHC staining for GRP78 (green) and p62 (red) in the dorsal horn from sham and SCI animals. Scale bars are 20 μm.
